# Exploring the role of ubiquitination modifications in migraine headaches

**DOI:** 10.3389/fimmu.2025.1534389

**Published:** 2025-01-31

**Authors:** Qian Zhu, Jin Yang, Lei Shi, Jieying Zhang, Peng Zhang, Junlong Li, Xiaoli Song

**Affiliations:** ^1^ First Teaching Hospital of Tianjin University of Traditional Chinese Medicine, Tianjin, China; ^2^ National Clinical Research Center for Chinese Medicine Acupuncture and Moxibustion, Tianjin, China

**Keywords:** ubiquitination, migraine, inflammation, biomarker, central sensitization, peripheral sensitization

## Abstract

Migraine is a complex neurovascular disorder whose pathogenesis involves activation of the trigeminal vascular system, central and peripheral sensitization, and neuroinflammation. Calcitonin gene-related peptide (CGRP) plays a dominant role and activation of MAPK and NF-κB signaling pathways regulates neuropeptide release, glial cell activation, and amplification of nociceptive signals. Aberrant activation of these pathways drives migraine onset and chronicity. The ubiquitin-proteasome system (UPS) is involved in neurological and inflammatory disorders. ubiquitination in the UPS is achieved through a cascade of enzymes, including Ub-activating enzyme (E1), Ub-coupling enzyme (E2), and Ub-ligase (E3). The aim of this review is to systematically explore the role of ubiquitination in the regulation of MAPK and NF-κB signaling pathways, with a focus on the mechanisms of ubiquitinating enzymes in neuroinflammation and pain signal amplification, and to explore their potential as diagnostics, biomarkers, predictors of response to therapy, and monitoring of chronicity in migraine disease.

## 1 Introduction

Migraine is now the sixth most prevalent disease worldwide and a leading cause of disability (1). According to the Global Burden of Disease Study (GBD) 2019, approximately 8-15% of migraine sufferers have at least one attack per year. The pathophysiology of migraine is thought to involve abnormal activation of the trigeminal vascular system, peripheral and central sensitization, and neuroinflammation (2). Among them, calcitonin gene-related peptide (CGRP) plays a dominant role. Related regulatory pathways, such as the mitogen-activated protein kinase (MAPK) and nuclear factor-kappa B (NF-κB) pathways, regulate neuropeptide release, glial cell activation, and pain signaling, mediating inflammatory and sensitizing responses. These may drive is the development of migraine pathogenesis and chronicity.

As a type of protein post-translational modification (PTM), the ubiquitin-proteasome system (UPS) is a pathway for protein ubiquitination and degradation. Ubiquitination involves a cascade reaction of three enzymes: Ub-activating enzymes (E1s), Ub-coupling enzymes (E2s) and Ub-ligases (E3s); deubiquitination is mediated by deubiquitinating enzymes (DUBs) (3, 4). Disturbances in the UPS have been shown to be associated with the induction and severity of a variety of neurologic and inflammatory disorders, suggesting its possible involvement in the pathogenesis of migraine headaches.

This paper systematically explores the role of ubiquitination in the regulation of MAPK and NF-κB signaling pathways, focusing on the regulatory mechanisms of ubiquitinating enzymes in neuroinflammation and nociceptive signal amplification, as well as exploring its feasibility as a biomarker for disease diagnosis, prediction of therapeutic response, and chronicity monitoring.

## 2 Pathogenesis of migraine and association of MAPK and NF-κB signaling pathways

**Figure 1:**
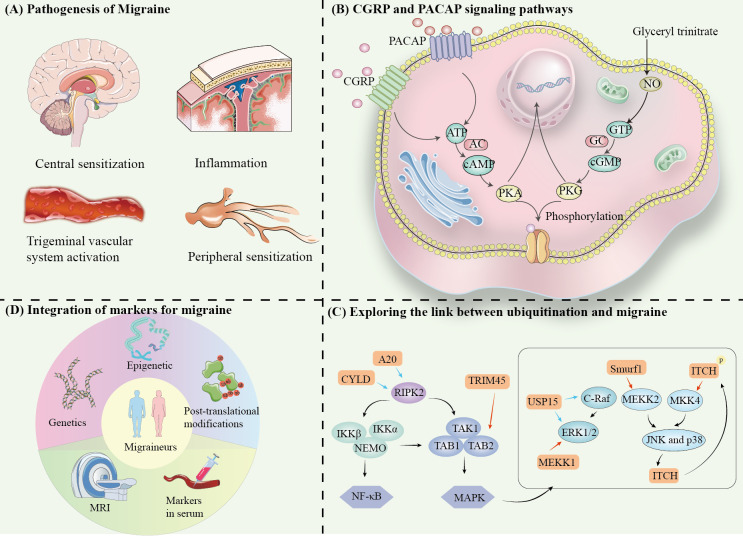
**(A)** Pathogenesis of Migraine. **(B)** CGRP and PACAP signaling pathways. **(C)** Exploring the link between ubiquitination and migraine. **(D)** Integration of markers for migraine.

Migraine is a complex neurovascular disorder whose pathogenesis involves multilevel interactions between the nervous and vascular systems. Recent studies have shown that the core mechanisms of migraine include activation of the trigeminal vascular system, central sensitization, peripheral sensitization and neuroinflammation. Together, these mechanisms form the pathophysiologic basis of migraine (**Figure 1A**).

### 2.1 Trigeminal vascular system activation

The trigeminal vascular system is the anatomical and physiological basis of migraine attacks, and a key mechanism of migraine nociceptive perception (5–7) (**Figure 1B**). Stimulation of the trigeminal ganglion (TG) leads to vascular release of neurotransmitters and inflammatory mediators, such as CGRP, PACAP, and substance P upon activation (8). These molecules bind to receptors and induce intracranial vasodilation, neuroinflammatory activation and enhanced pain signaling. Then, it leads to sensitization of secondary neurons in the trigeminal cervical complex (TCC) of the brainstem, and ultimately activate tertiary neurons in the thalamus (9).

CGRP plays a key role in neural sensitization and amplification of pain signals in migraine by binding to its receptor, which consists of CLR and RAMP1. After binding, CGRP activates Gs proteins, initiating the production of cAMP by adenylyl cyclase (AC). It further activates protein kinase A (PKA), which regulates sodium and potassium ion channel (10–12). Currently, CGRP receptor antagonists (gepants) have become effective acute treatments (13, 14).

PACAP acts mainly by binding to PAC1, VPAC1 and VPAC2 receptors (15–18). Similar to CGRP, PACAP also activates the cAMP-PKA pathway leading to migraine headaches (19). In addition, PACAP appears to have unique downstream effects, such as acting through direct activation of the EPAC pathway (20, 21). Furthermore, through the Ras-Raf-MEK-ERK signaling pathway, PKA contributes to extracellular signal-regulated kinase (ERK) phosphorylation and activation, which in turn combine peripheral sensitization and central sensitization mechanisms (15, 22).

### 2.2 Peripheral sensitization

Peripheral sensitization refers to a decreased threshold of damage receptors and an increased sensitivity of injury receptors. In response to repetitive stimuli or inflammatory states, excitability of sensory neurons in the periphery increases, which enhances the transmission of pain signals (23–25).

Transient receptor potential (TRP) channels, such as TRPV1, TRPA1 and TRPM8, have been shown to be closely associated with neuropathic pain. Studies have shown that blocking TRPV1 and TRPA1 significantly attenuates neurogenic hypersensitivity reaction (26–31).

In the inflammatory state, TRPV1 receptors are activated, triggering a massive inward flow of sodium and calcium ions, which triggers depolarization of injury receptors, thereby amplifying pain signals (32). Activation of TRPV1 also stimulates the release of CGRP and substance P from the nerve endings, leading to vasodilation (33).Vasodilation leads to an increase in the mechanical pressure on the local tissues, thereby further stimulating the sensory neurons of the trigeminal nerve fiber system, causing a decrease in the nociceptive threshold of injury receptors.

Notably, TRPV1 not only causes acute pain, but also persistent pain, especially pain associated with inflammation (34–36). It was found that hormone level changes may indirectly regulate the release and physiological roles of CGRP by affecting the expression and activity of TRPV1 (37). In particular, TRPV1 activation not only promotes CGRP release, but also enhances synaptic transmission efficiency and exacerbates central sensitization of neurons by promoting the release of glutamatergic vesicles (38).

Through the cAMP-PKA pathway, CGRP modulates ion channels on neuronal cell membranes, enhancing peripheral nerve sensitivity. Upon tissue injury, CGRP acts synergistically with substance P, leading to an increase in vascular permeability of local tissues and the release of inflammatory factors (39–42). Inflammatory response further activates primary afferent neurons, forming a positive feedback loop that amplifies pain signals through inflammation.

### 2.3 Central sensitization

Central sensitization is an important mechanism of chronic pain and is characterized by CNS hyperresponsiveness to injurious stimuli (43–45). It involves increased presynaptic neurotransmitter release and persistent neuroinflammation (45–47). Enhanced synaptic transmission in the caudate of the TNC is the neural basis for central sensitization in a CM rat model (45, 48, 49). The release of glutamate (Glu) is a key step in central sensitization, the regulation of which is dependent on the activation of ERK and p38 signaling pathways (50, 51). At presynaptic sites, the central terminals of injurious primary afferent nerves, activation of cytokine receptors and chemokine receptors leads to phosphorylation of ERK and p38 (P-ERK, P-p38) and glutamate (Glu) release. This synaptic vesicle release is associated with activation of the ion channels TRPV1, Na channel (52, 53).

At postsynaptic sites, phosphorylation of AMPA and NMDA glutamate receptors significantly enhances neuronal responsiveness to excitatory signals. Phosphorylation of AMPA receptors by PKA)and Ca^2+^/calmodulin-dependent kinase II (CaMKII) results in increased insertion into synaptic membranes, a significant elevation in open probability, and enhanced response to glutamate response (54, 55). phosphorylation of NMDA receptors in response to Src kinase enhances calcium ion permeability and amplifies postsynaptic Ca^2+^ signaling (56, 57).

In addition, P-ERK reduces the repolarization capacity of postsynaptic neurons by inhibiting their potassium channel activity, leaving them in a state of hyperexcitability (58, 59). Notably, P-ERK translocates to the nucleus and promotes the phosphorylation of the cAMP response element-binding protein (CREB), which activates proinflammatory factors, such as c-Fos and NK-1, as well as the nociceptive regulation-related gene expression, further consolidating the molecular basis of central sensitization (60, 61).

### 2.4 Neuroinflammation

By activating the NF-κB signaling pathway, microglia and astrocytes are activated in response to injurious stimuli and release a series of proinflammatory factors and chemokines, including tumor necrosis factor (TNF-α), interleukin-1β (IL-1β), and chemokines (62–64). Release of these factors enhances the inflammatory response and further exacerbates neuronal excitability. It was shown that upregulation of miR-155-5p activated the NF-κB signaling pathway by inhibiting SIRT1, which in turn exacerbated the release of microglial pro-inflammatory factors and neuroinflammation by inhibiting miR-155-5p, it was able to activate SIRT1 in the TNC region of CM mice, which effectively reduced neuroinflammation (60). In addition, activation of NLRP3 inflammatory vesicles was also involved in the release of pro-inflammatory factors, which further contributed to the onset of central sensitization and the chronicity of migraine (65, 66).

Further studies revealed that autophagy plays an important role in the regulation of neuroinflammatory and oxidative stress processes in astrocytes. By inhibiting autophagy, the binding of TRAF6 to K63 ubiquitinated proteins could be promoted, which increased the activities of p-MAPK8/JNK and NF-κB, thereby exacerbating the release of pro-inflammatory factors (e.g., TNF-α, IL-1β). Conversely, activation of autophagy can significantly reduce neuroinflammatory levels (67).

## 3 Ubiquitination and migraine

Multiple family and twin studies have shown that common migraine is heritable, with heritability estimates ranging from 30% to 60%, suggesting the presence of genetic factors that predispose individuals to migraine (68, 69). Migraine has a heritability of 42%, and relatives of people with migraine are 2- to 3-fold more likely to have the disorder (70). Although the risk of migraine is predominantly polygenic, pathogenic variants in a single gene can lead to monogenic migraine disorders (e.g., familial hemiplegic migraine FHM) and the gene is dominant, this suggests that the susceptibility and complexity of migraines may be based on genetics and may be subject to different gene-gene and gene-environment interactions (71).

The susceptibility and pathophysiological aspects of migraine have been explained from a locus perspective, but recently there has been a developing interest in investigating the role of gene regulatory mechanisms in the predisposition and chronicity of migraine, particularly epigenetic regulation. The number of studies on the role of epigenetic mechanisms in migraine is now found to be increasing yearly. Epigenetic mechanisms regulate cell cycle development by controlling the expression of individual genes, including acetylation, phosphorylation, etc (72, 73). TRP channels can convert injurious stimuli into pain signals, and the expression of TRPA1, TRPA1 encoding gene, has been demonstrated to be affected by pain-related syndromes. Acetylation modifications, this process may enhance neural excitability and facilitate pain transmission by altering electrical activity or localization (74, 75).

Similar to acetylation, ubiquitination also acts at the protein level. Ubiquitination occurs by adding single or multiple ubiquitin molecules to a target protein, modulating its stability, activity, or degradation. Studies demonstrating the relevance of the ubiquitination system to migraine are extremely limited. Ubiquitin C terminal hydrolase 1 (UCHL1), an enzyme with both ligase and hydrolase activities, is present in almost all neurons (76, 77). Serum levels of UCHL1 were significantly elevated during acute attacks in migraine patients; also, before treatment, UCHL1 levels were significantly and positively correlated with visual analog scores (VAS) (78). This suggests that UCHL1 can be used to assess seizure severity and response to treatment. Although there is conclusive evidence providing the relevance of ubiquitination system-associated proteins in acute attacks of migraine, there is little direct evidence that the meso-ubiquitination system plays a role in migraine.

Ubiquitinating enzymes (e.g., MEKK1, Smurf1, ITCH, and TRIM45) and deubiquitinating enzymes (e.g., USP15, A20, and CYLD) may be involved in the occurrence and chronicity of migraine by regulating the ubiquitination of MAPK, JNK, and NF-κB signaling pathways.

Migraine mechanisms involve dynamic processes of central sensitization and peripheral sensitization, abnormal activation of the trigeminal-vascular system, and neurogenic inflammation. Each of the four mechanisms is associated with various enzymes of the ubiquitination system, given the important role of ubiquitination in other neuroinflammatory disorders, it is reasonable to hypothesize that ubiquitination is involved in migraine attacks and chronicity (79–81).

Various trigger molecules can induce migraine, including CGRP, PACAP, adenosine triphosphate-sensitive potassium (KATP) channel opener, and large conductance calcium-activated potassium (BKCa) channel opener. The epigenetic link of CGRP and its potential in migraine has been discussed (82). In isolated trigeminal ganglion neurons, CGRP stimulates pain-related intracellular signaling molecules such as cAMP, CREB, MAPK, p38, and ERK (83). In the following, we explore the ubiquitylation of these proteins to play a function in migraine Possibilities (**Figure 1C**).

### 3.1 Regulation of ERK1/2 by ubiquitination

The ERK1/2 signaling cascade was first identified in four MAPK signaling pathways (84, 85). ubiquitination of ERK1/2 is regulated by MEKK1, which has a RING finger structure and exhibits an E3 ligase function, and USP15, a deubiquitinating enzyme. ERK2 is deubiquitinated by USP15, but the stability of the protein is not affected. Not only associated with ubiquitination, USP15 also induces ERK1/2 phosphorylation (86). Interestingly, USP15 is also known to regulate C-Raf DUB, binding to C-Raf and protecting the protein from proteolytic degradation by the 26S proteasome 34688658. overexpression of C-Raf and activation of the ERK1/2 signaling pathway cause overexpression of USP15 expression, leading to cell proliferation and migration (87).

### 3.2 Regulation of p38, JNK by ubiquitination

Although MEKK1 has ubiquitinating enzyme properties, it is still a member of the MAP3K family. jNK1/2/3 and p38 signaling cascades share upstream regulators such as MEKK1-4 and MKK4. In the inflammatory response, the E3 ubiquitin ligase Smad ubiquitination regulatory factor 1 (Smurf1) ubiquitinates the K48-conjugated polyubiquitin chain of MEKK2, the same type of ubiquitinating enzyme, ITCH, participates in a negative feedback loop of JNK (88). ITCH regulates MKK4 (89). ITCH is a downstream substrate of JNK, and activation of JNK promotes ITCH phosphorylation, and phosphorylated ITCH induces ubiquitination of K140 and K143 of MKK4 (90).

### 3.3 Regulation of NF-κB by ubiquitination

RIPK1 is the first kinase found in the RIPK family (91). RIPK2 does not have any death structural domains and does not trigger cell death signaling, but has a cysteine asparaginase activating and recruiting structural domain (CARD) that contributes to function in the NOD-like receptor (NLR)-associated inflammatory signaling pathway (92, 93). K63 ubiquitination of RIPK2 interacts with LUBAC and the kinase complex TAK1 and promotes linear ubiquitination of RIPK2, initiating the MAPK signaling pathway (94). Interestingly, the kinase complex also triggers a separate pathway for activation of the IKK complex, which consists of NEMO, IKKα, and IKKβ, and activation of the protein complex leads to the activation of NF-kinase. The IKK complex consists of NEMO, IKKα, and IKKβ, and activation of this protein complex leads to NF-κB activation. In addition, deubiquitinating enzymes A20 and CYLD have linear bonding specificity that can counteract RIPK2 ubiquitination (95, 96). Linear ubiquitination is much more attractive for NEMO binding than normal polyubiquitination, so ubiquitinated RIP1 also attracts NEMO/IKK and TAB/TAKI bindings, and thus activates downstream NF-KB signaling pathways. NF-KB signaling pathway and JNK, P38/MAPK signaling pathway (97).

The E3 ligase tripartite motif-containing 45 (TRIM45) constitutively interacts with TAB2 and promotes polyubiquitination of the TAB2-Lys-63 linkage, leading to the formation of the TAB1-TAK1-TAB2 complex and activation of TAK1, and ultimately the activation of the nuclear factor-carbamyl B (NF-κB) signaling pathway (98).

## 4 The potential of ubiquitination in migraine treatment

In recent years, the development of drugs targeting ubiquitinating and deubiquitinating enzymes has emerged as a research hotspot in the field of precision therapeutics (99). Although most studies have focused on cancer, the role of these drugs in ubiquitination regulation provides a potential reference for migraine treatment.

Inhibitors of ubiquitinating enzymes (TAK-243, an E1 ligase inhibitor, and MLN4924, an E3 ligase inhibitor) as potential drugs for precise regulation of inflammatory responses and cell signaling pathways (100–102).

The development of activators of USP family deubiquitinating enzymes could be a potential strategy in the treatment of migraine. USP25 inhibits the overactivation of the NF-κB and MAPK pathways by removing the K63 polyubiquitin chain on TAB2, thereby attenuating microglia-mediated neuroinflammation (103). Notably, it has been found that USP5 inhibits the expression of proinflammatory factors by maintaining NF-κB signaling pathway activation to promote the expression of pro-inflammatory factors, whereas its inhibitor Vialinin A was able to significantly reduce TNF-α and IL-1β-induced pro-inflammatory gene expression, suggesting that USP5 inhibitors may serve as drug candidates for the treatment of inflammatory diseases, such as Kawasaki disease (104). In addition, the metalloproteinase inhibitor THL significantly inhibited NLRP3 activation by blocking BRCC3 complex-mediated deubiquitination, alleviating inflammation and tissue damage in a variety of inflammatory disease models such as sepsis, autoimmune encephalomyelitis, and nonalcoholic fatty liver disease (105).

## 5 Conclusion

In recent years, studies on migraine mechanisms have revealed the important roles of inflammatory factors, neurogenic inflammation, and CGRP signaling pathways in the disease. As one of the important mechanisms of epigenetic regulation, ubiquitination plays a key role in the pathogenesis and chronicity of migraine by regulating protein degradation, cell signaling, and inflammatory responses. In terms of genetic markers, mutations in CACNA1A, ATP1A2, and SCN1A are closely associated with FHM, while variants in NOTCH3 and TREX1 genes have been linked to migraine and its associated cerebrovascular diseases (e.g., cerebral arteriolar dominant disorders) (106–109). In addition, DNA methylation analysis identified methylated regions specific to migraine patients, suggesting a role for epigenetic modifications in migraine susceptibility (110). In the blood, plasma CGRP levels were significantly elevated in patients during migraine attacks (111). On the imaging side, functional magnetic resonance imaging (fMRI) and diffusion tensor imaging (DTI) studies have shown that migraine patients have increased visual Increased thickness of the visual cortex, significantly altered functional connectivity in nociceptive pathways, and white matter hyperintensity are characteristic imaging hallmarks of migraine with aura (112, 113). These studies suggest the importance of plastic changes in neural structure and function in migraine.

Future studies need to further elucidate the specific link between ubiquitination and the above markers. For example, do ubiquitination modifications affect the degradation of key molecules in the CGRP signaling pathway? Does it mediate the chronicity of migraine by modulating inflammatory factors such as TNF and IL-6? By integrating multi-modal data from epigenetics, genetic markers, blood biomarkers, and neuroimaging, we aim to enhance the understanding of the molecular mechanisms underlying migraine, thus advancing marker-based precision medicine. This integrated approach may pave the way for more effective, individualized treatments for migraine patients (114) (**Figure 1D**).
